# Characterization of the hepatitis B virus DNA detected in urine of chronic hepatitis B patients

**DOI:** 10.1186/s12876-018-0767-1

**Published:** 2018-03-16

**Authors:** Surbhi Jain, Ying-Hsiu Su, Yih-Ping Su, Sierra McCloud, Ruixia Xue, Tai-Jung Lee, Shu-Chuan Lin, Selena Y. Lin, Wei Song, Jamin D. Steffen, Chi-Tan Hu

**Affiliations:** 1 JBS Science, Inc., Doylestown, PA 18902 USA; 2 The Baruch S. Blumberg Institute, Doylestown, PA 18902 USA; 3 The Second Hospital of Yuncheng, Yuncheng, Shanxi Province 044000 China; 4 Buddhist Tzu Chi General Hospital and Tzu Chi University, 707, Sec. 3, Chung-Yang Rd, Hualien, 970 Taiwan, R.O.C.; 5 U-Screen Dx Inc., Doylestown, PA 18902 USA; 6 Hepron Molecular Lab, Inc., Doylestown, PA 18902 USA

**Keywords:** Hepatitis B virus, Hepatitis B virus DNA, Urine, Infection, HBV DNA PCR assays

## Abstract

**Background:**

Detection of human hepatitis B virus (HBV) DNA in the urine of patients with chronic hepatitis B infection (CHB) has been reported previously, suggesting urine could provide a potential route of horizontal HBV transmission. However, it is not clear whether the HBV DNA detected in urine is indeed full-length, infectious viral DNA. The aim of this study is to assess the potential infectivity of urine from patients with CHB and to correlate HBV DNA detection in urine with clinical parameters, such as serum viral load and HBeAg status.

**Methods:**

Urine from 60 CHB patients with serum viral loads ranging from undetectable to 10^8^ IU/mL were analyzed for HBV DNA and serum immune markers. HBV DNA was detected from total urine DNA and size-fractionated urine DNA (separated into ≤1 kb and > 1 kb fractions) by PCR analysis of six regions of the HBV genome.

**Results:**

Twenty-seven of 59 (45.7%) patients with HBV serum viral load (≥20 IU/mL) contained at least 20 copies per mL of fragmented HBV DNA in urine detected in at least 1 of the 6 PCR assay regions. Only one patient contained HBV DNA detected by all six regions, and was found to have evidence of blood in the urine. Sixteen of 25 urine samples with high viral load (> 10^5^ IU/mL) and 11 of 34 urine samples with low viral load (< 10^5^ IU/mL) contained detectable HBV DNA. Twelve of 27 (44.44%) patients with detectable HBV DNA in urine were HBeAg positive, and only 5 of these HBeAg positive patients were in the group of 33 (15.15%) patients with no detectable HBV DNA in urine. By Fishers’ exact test, HBV DNA in urine is significantly associated with high serum viral load (*P* = 0.0197) and HBeAg (*P* = 0.0203).

**Conclusions:**

We conclude that urine from CHB patients with healthy kidney function should not contain full-length HBV DNA, and therefore should not be infectious.

**Electronic supplementary material:**

The online version of this article (10.1186/s12876-018-0767-1) contains supplementary material, which is available to authorized users.

## Background

Human hepatitis B virus (HBV) infection affects primarily the liver, which can result in chronic HBV infection (CHB) and a heightened risk for cirrhosis and liver cancer. Despite the availability of a vaccine, HBV infection remains a major global health problem affecting about 240 million people worldwide [1–7]. In highly endemic areas, HBV is commonly transmitted perinatally or through horizontal transmission via exposure to infected blood. HBV can also spread by percutaneous or mucosal exposure to infected body fluids [2–8].

We have previously reported the detection of HBV DNA in the urine of patients with CHB [9] along with others [8, 10–14], suggesting urine from CHB patients could be a potential route of horizontal and nosocomial transmission. We and others have shown that urine contains fragmented DNA from circulation [9, 15–18]. Circulating cell-free DNA is known to derive from apoptosis or necrosis of host cells [17, 19–21]. It is not clear whether HBV DNA detected in urine consists of the full-length 3.2 kb viral DNA (a potential indicator of infectious virus), or is merely fragmented HBV DNA derived from the infected liver. Detection of short regions of HBV DNA by PCR cannot discriminate between fragmented and full-length DNA. Therefore, a more thorough understanding of the HBV DNA detected in urine is important to better provide proper handling guidelines of urine samples from HBV infected patients.

In this study, we evaluate the nature of the DNA detected in urine from patients with CHB to assess the potential infectivity and to correlate HBV DNA detection in urine with clinical parameters, such as serum viral load and HBeAg status.

## Methods

### Study subjects

Urine and blood samples were obtained with written informed consents from 60 patients with CHB and were acquired under institutional review board approvals from the Buddhist Tzu Chi Medical Center in Hualien, Taiwan. Detailed patient information is provided in Table 1 and summarized in Table 2. We excluded patients with autoimmune diseases, which might have caused immune complex deposition to compromise renal filtration function.

**Table 1:** Detailed clinicopathological characteristics of the patient population

Sample ID	Age	Gender	Liver Disease		Serum Analysis						Urine analysis		Any known kidney disease	Anti-viral therapy
				HBV viral load category	HBV viral Load (IU/mL)	HBeAg (+/−)	HBsAg (+/−)	AST (IU/L)	ALT (IU/L)	AFP (ng/ml)	RBC/ High power field	Urine albumin (mg/dL)		
1	29	F	Cirrhosis	High	1.70E + 08	+	+	44	36	< 1.3	2~ 5	30	–	No
2	36	F	CHB	High	1.70E + 08	+	+	20	17	1.7	0	–	CKD	No
3	32	M	CHB	High	1.70E + 08	+	+	33	64	1.3	0~ 2	–	–	No
4	41	F	Cirrhosis	High	1.34E + 08	+	+	191	126	5.8	2~ 5	–	–	Yes
5	52	F	CHB	High	6.07E + 07	+	+	23	21	4	0~ 2	10	FSGS,CGN	No
6	52	M	Cirrhosis	High	5.81E + 07	+	+	109	48	81	0~ 2	–	–	Yes
7	68	F	Cirrhosis	High	4.57E + 07	+	+	73	38	3.6	2~ 5	200	–	Yes
8	29	F	CHB	High	4.53E + 07	+	+	48	60	1.8	0~ 2	–	–	No
9	32	M	CHB	High	4.13E + 07	+	+	394	921	16.4	0~ 2	–	–	Yes
10	22	M	CHB	High	3.04E + 07	+	+	160	556	5.2	0	–	–	No
11	54	M	CHB	High	1.44E + 07	–	+	24	25	2.8	0	–	–	No
12	72	F	CHB	High	3.89E + 06	–	+	58	62	35.1	0~ 2	–	–	No
13	59	M	CHB	High	1.78E + 06	–	+	69	97	4.3	0~ 2	–	–	No
14	65	M	CHB	High	1.37E + 06	–	+	60	110	5	0~ 2	–	–	No
15	60	F	CHB	High	8.30E + 05	–	+	171	242	2.8	0~ 2	–	–	Yes
16	42	M	CHB	High	7.47E + 05	–	+	22	24	< 1.3	2~ 5	–	–	Yes
17	55	F	CHB	High	7.25E + 05	+	+	299	424	7.4	0~ 2	–	–	Yes
18	60	M	Cirrhosis	High	4.17E + 05	–	+	64	63	32.8	0~ 2	–	–	No
19	36	M	CHB	High	3.38E + 05	+	+	34	50	4.2	2~ 5	10	–	Yes
20	60	F	CHB	High	3.19E + 05	–	+	99	124	2.6	0~ 2	–	–	No
21	41	M	CHB	High	2.10E + 05	–	+	89	207	6.5	0	–	–	No
22	48	M	CHB	High	1.67E + 05	–	+	31	35	< 1.3	0	–	–	No
23	63	F	CHB	High	1.48E + 05	–	+	43	30	1.9	0~ 2	–	–	No
24	65	F	HCC	High	1.08E + 05	+	+	186	95	2623.1	0~ 2	–	–	No
25	62	M	CHB	High	1.02E + 05	–	+	27	39	3.4	0~ 2	–	–	No
26	48	F	CHB	Low	9.31E + 04	–	+	28	31	3.3	0~ 2	–	–	No
27	46	M	CHB	Low	4.85E + 04	–	+	33	53	2.1	0	–	–	No
28	67	F	CHB	Low	3.93E + 04	–	+	38	27	5	0	–	–	No
29	52	M	CHB	Low	3.82E + 04	–	+	32	37	4.1	0~ 2	–	–	No
30	75	F	CHB	Low	2.75E + 04	–	+	41	30	3.4	2~ 5	–	–	Yes
31	31	M	CHB	Low	2.65E + 04	+	+	38	57	< 1.3	0	–	–	Yes
32	44	M	Cirrhosis	Low	1.74E + 04	+	+	72	95	2.4	> 100	70	–	No
33	48	F	CHB	Low	1.65E + 04	–	+	22	22	< 1.3	0	–	–	No
34	54	M	CHB	Low	1.49E + 04	–	+	40	52	< 1.3	0~ 2	10	–	No
35	51	F	CHB	Low	1.01E + 04	–	+	36	22	1.4	2~ 5	–	NA	No
36	48	M	CHB	Low	9.68E + 03	–	+	38	30	6.7	0~ 2	600	–	No
37	53	F	CHB	Low	6.40E + 03	–	+	46	81	2.1	0~ 2	–	–	No
38	59	M	CHB	Low	4.36E + 03	–	+	44	25	2.1	0~ 2	–	–	Yes
39	46	M	CHB	Low	4.08E + 03	–	+	27	35	5.2	0	–	–	No
40	18	M	CHB	Low	3.18E + 03	+	+	45	78	1.6	0~ 2	10	–	No
41	57	M	CHB	Low	3.00E + 03	–	+	29	38	3.5	0~ 2	–	–	Yes
42	57	F	Cirrhosis	Low	2.93E + 03	–	+	33	44	1.4	0~ 2	–	–	No
43	28	M	CHB	Low	2.68E + 03	–	+	35	48	21.4	0~ 2	30	–	No
44	47	M	CHB	Low	2.42E + 03	–	+	42	82	2.1	2~ 5	10	–	No
45	64	F	CHB	Low	2.24E + 03	–	+	58	67	3.9	0~ 2	–	–	No
46	58	M	CHB	Low	2.02E + 03	–	+	26	28	< 1.3	0~ 2	20	–	No
47	43	F	CHB	Low	1.83E + 03	–	–	26	26	6.2	2~ 5	–	–	No
48	56	F	CHB	Low	1.50E + 03	–	+	26	23	2.4	0~ 2	–	–	No
49	33	F	CHB	Low	1.49E + 03	–	+	28	42	3.2	0~ 2	–	–	No
50	56	F	CHB	Low	1.25E + 03	–	+	34	67	2.6	2~ 5	–	–	Yes
51	38	M	CHB	Low	1.17E + 03	–	+	43	91	5	0~ 2	–	–	No
52	38	M	CHB	Low	1.09E + 03	–	+	38	77	2.4	0~ 2	10	–	No
53	61	M	CHB	Low	1.07E + 03	–	+	26	30	2.1	0~ 2	–	–	No
54	51	M	CHB	Low	1.06E + 03	–	+	21	27	2.8	0	–	–	No
55	28	F	CHB	Low	3.41E + 02	–	+	36	32	2.7	0~ 2	–	–	Yes
56	34	M	CHB	Low	5.40E + 01	–	+	32	53	2.4	0	–	–	Yes
57	36	M	CHB	Low	3.20E + 01	+	+	116	98	3.5	2~ 5	50	–	Yes
58	69	M	HCC	Low	2.00E + 01	–	+	45	21	5060.9	> 100	30	–	No
59	40	M	CHB	Low	2.00E + 01	–	–	41	65	3.8	0	–	–	Yes
60	38	M	CHB	Low	< 20	–	+	46	57	7.7	0~ 2	–	–	Yes

*F* female, *M* male, *CHB* Chronic hepatitis B infection, *NA* Data not available, *CKD* chronic kidney disease, *FSGS* focal segmental glomerulosclerosis, *CGN* chronic glomerulonephritis

For all patients who received antiviral treatment, the drug received was “Telbivudine,” which has no known renal side effects

**Table 2:** Summary of clinicopathological characteristics of the patient population

Mean Age ± Std Dev (years)	48.45 ± 13.33
Gender (M/F)	35/25
Liver disease (Chronic Hepatitis B/ HBV Cirrhosis/ HBV-HCC)	51/7/2
Serum viral load (Range, IU/L)	< 20–1.7 E + 08
HBeAg (Positive/Negative)	17/43
HBsAg (Positive/Negative)	58/2
Mean AST levels ± Std Dev (IU/L)	62.2 ± 66.85
Mean ALT levels ± Std Dev (IU/L)	86.75 ± 141.29
Antiviral therapy (Yes/No)	18/42

### Blood and urine analyses

Blood samples were collected immediately following urine sample collection. Sera were examined for alanine transaminase (ALT), aspartate (AST) transaminase, HBV viral load (Roche Molecular Diagnostics, USA), Hepatitis B envelope antigen (HBeAg), and Hepatitis B surface antigen (HBsAg). Alpha-fetoprotein and C-reactive protein (CRP) in blood were also determined by high sensitivity CRP (Dade Behring Inc., Marburg, Germany). The presence of antibodies directed against nuclear antigens in urine was examined using the ANA kit (Antibodies Incorporated, Davis, California). The levels of rheumatic factor were examined using the Rheumatoid Factor ELISA Kit, (Hycor Biomedical, California, United States). The levels of cryoglobulin were examined using the Cryoglobulin ELISA Kit (Cryoglobulin ELISA Kit, MyBioSource, California, United States). The levels of serum immunoglobulins were examined using the Immunoglobulin kit (Dade Behring Inc., Marburg, Germany). The levels of viral markers, including CMV IgM and IgG, EB-VCA IgM and IgG, EBEA and EBNA, and HSV IgM were examined using the Euroimmun ELISA kit (Euroimmun, Luebeck, Germany). The presence of red blood cells in urine was confirmed by examination under the microscope.

### Urine collection, DNA isolation, and low molecular weight urine DNA fractionation

Freshly collected urine was immediately mixed with 0.5 mol/L EDTA, pH 8.0, to a final concentration of 10 mmol/L EDTA and stored at − 20 °C. Total urine DNA was isolated by adding an equal volume of 6 mol/L guanidine thiocyanate (Sigma, St. Louis, MO) to thawed urine as described previously [17]. The fractionated high molecular weight (HMW) urine DNA (> 1 kb) and low molecular weight (LMW) urine DNA (≤1 kb) was obtained from total urine DNA using carboxylated magnetic beads (Agentcourt Bioscience Corporation, Beverly, MA), as previously described [22].

### Quantification of DNA

**Fig. 1:**
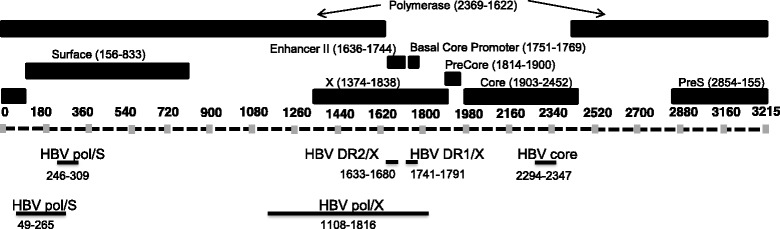
Diagram of the HBV genome (NC_003977.1), indicating location of primers and the amplicons generated by qPCR assays in this study. Black rectangles represent the following HBV regions: polymerase, enhancer II, basal core promoter, precore, Surface, X, Core, and pre-S gene. These regions correspond to the dashed line representing the HBV genome with vertical gray bars indicating nucleotide location. The black lines below this HBV genome map indicate the amplicon location of each qPCR assay used in the study. The name of the region targeted by the qPCR assay is written above the black line and the exact location of the amplicon is indicated below the black line

HBV DNA was quantified using the HBV DNA quantification kit (JBS Science, Inc. Doylestown, PA) according to the manufacturer’s specifications. The schematic diagram of the region of each PCR assay used in this study in the HBV genome is illustrated in Fig. 1. The HBV DNA quantification kit (JBS Science Inc., Doylestown, PA) consists of five quantitative PCR assays that provide quantification of four different regions of the HBV genome (NC_003977·1): The HBV polymerase /Surface antigen (pol/S, nt. 246–309 and nt. 49–265), HBV DR2/X (nt. 1633–1680), HBV DR1/X (nt. 1741–1791), and HBV core (nt. 2294–2347). Note the limit of detection (LOD) is 10 copies per reaction for HBV pol/S (nt. 246–309), DR2/X, DR1/X, and HBV core assays, and 50 copies per reaction for the HBV pol/S (nt. 49–265) assay, as determined by the standards per manufacturer’s specification. The existence of DNA containing 709 bp of the HBV pol/X region (nt. 1108–1816) was determined by an end-point PCR assay. The end-point PCR reactions were set up with 1 × Qiagen PCR buffer, 250 μM deoxyribonucleotide triphosphate mix, 1.0 μmol/L primers (F:TTCTCGCCAACTTACAAGGC, R:AAAAAGTTGCATGGTGCTG), Hotstart Taq Polymerase Plus (Qiagen, Valencia, CA), at 95 °C for 5 min, then (95 °C for 30 s, 56 °C for 30 s, 72 °C for 30 s) for 40 cycles. The input of isolated DNA for each assay was from 0.5 mL urine and was performed in duplicate. Quantification of human genomic DNA was carried out using the *TP53* DNA quantification assay as described previously [18].

### Statistical analysis

The association of HBV DNA in urine with serum viral load and HBeAg were analyzed by Fisher’s exact test. Kruskal-Wallis test was performed to determine the correlation between urinary HBV DNA and age, gender, and AST or ALT levels. All statistical tests were performed using SPSS Statistics 20 (IBM, Armonk, NY) and QuickCals (GraphPad Software, La Jolla, CA).

## Results

### Characterization of the study population

Previous studies have suggested that highly viremic HBV carriers may have high titers of HBV DNA in body fluids other than blood, such as urine [13, 14]. In order to investigate whether urine from patients with high viremia contains infectious HBV, we analyzed 25 urine samples from patients that have viral loads ranging from 10^5^ to 10^8^ IU/mL, designated as the “high viral load” group. In addition, we analyzed urine from 35 CHB patients whose viral loads were below 10^5^ IU/mL, designated as the “low viral load” group, as summarized in Table 1 (listed in descending order of their serum HBV viral load). Interestingly, Sample ID #59 was negative for surface antigen with a serum viral load of 20 IU/mL, suggesting an occult HBV infection. The clinicopathological characteristics of the patient population are summarized in Table 2. The mean age of the study population was 48.8 years (SD ± 13.2), consisting of 35 males and 25 females. Seven of the 60 CHB patients had Child Pugh A liver cirrhosis, and two of them were known to have hepatocellular carcinoma. Biomarkers with tested values above the normal range (positive) for any individual in this study cohort are included in Table 1. Immune disease markers (Antinuclear antibody, Rheumatic factor, Cryoglobulin, IgM, IgA, IgE, IgG and IgG4), viral markers (CMV IgM and IgG, EB-VCA IgM and IgG, EBEA and EBNA, HSV IgM) and inflammatory marker (C-reactive protein) were found to be negative in all patients of this study cohort, which suggests this study population has no individual with detectable immune complex deposition.

### Detection of HBV DNA in urine of patients with chronic hepatitis B

We employed five quantitative PCR (qPCR) assays and one end-point PCR assay for four different regions of the HBV genome (Fig. 1 and Additional file 1). Our hypothesis was that if the HBV DNA in urine is full-length (3.2 kb genome), any HBV PCR assay should be able to produce amplified product predominantly in both total urine DNA and size-fractionated HMW DNA (> 1 kb). In contrast, if the HBV DNA detected is mostly fragmented, it should be detected predominantly in both total urine DNA and size-fractionated LMW DNA (≤1 kb).

**Fig. 2:**
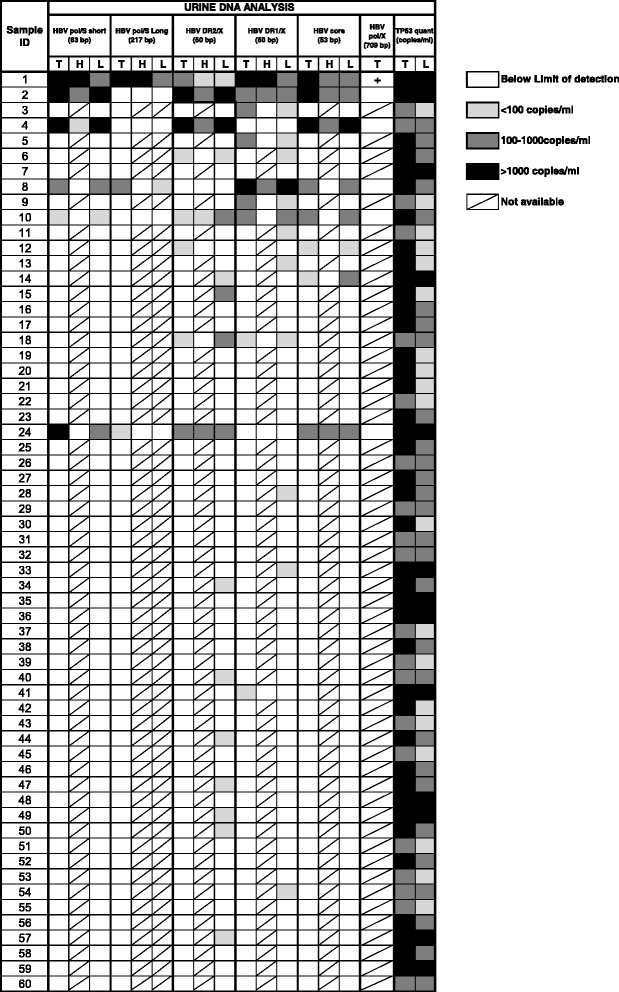
Summary of urine DNA analysis by PCR assays targeting multiple locations in the HBV genome. The amount of DNA detected is indicated by patterned boxes as follows: empty box: below the limit of detection; light gray box: less than 100 copies/ml; dark gray box: 100–1000 copies/ml; black box: greater than 1000 copies/ml; box with a slash: Not available, the assay was not run. T: Total urine DNA, H: High molecular weight urine DNA fraction, L: low molecular weight urine DNA fraction

We first performed the five HBV quantitative PCR assays on total urine DNA from all 60 CHB patients. For samples positive for HBV DNA by at least 3 assays, we performed an end-point PCR assay that generates a much larger amplicon (709 bp) in the HBV polymerase/X region (nt. 1108–1816). For samples detecting HBV DNA by at least 1 assay, we performed a fractionation of the total urine DNA into HMW and LMW urine DNA. Both fractions were then retested to determine if the HBV detected was of a full-length or fragmented nature. To control for the amount of DNA in urine, the total DNA was quantified by the *TP53* gene, as described previously [18]. The results are organized into four categories based on the quantities of DNA detected in Fig. 2 (illustrated by four patterns).

Thirteen of the 60 patient urine samples tested (Pt. IDs: 1–6, 8–10, 12, 14, 18, and 24) contained detectable HBV DNA in total urine DNA. Six of these patients (Pt. IDs: 1, 2, 4, 8, 10, and 24) contained HBV DNA detected by at least three of the five HBV DNA qPCR assays and only one (Pt. ID #1) produced the 709 bp PCR product, suggesting that the urine from this patient contained full-length HBV DNA. The HBV DNA in the remaining 12 patients is likely all fragmented, unless the amount of full-length DNA is below the limit of detection (20 copies per mL of urine).

To further investigate the size of the HBV DNA detected in the total urine DNA, we performed HBV DNA analysis in both fractionated HMW DNA and LMW DNA. As expected, only the HMW DNA from Pt. ID #1 was found to contain HBV DNA detected by all six assays, including the end-point PCR assay generating a 709 bp PCR product. Note, this patient contained very high serum viral load (10^8^ IU/mL) and her clinicopathological information of hematuria and albuminuria (red blood cells and serum albumin in urine) indicated a compromised renal barrier with contamination of blood in urine. Data obtained from the analysis of total urine DNA indicated that fragmented HBV DNA was unevenly distributed across the HBV genome. Among these 13 samples, HBV DNA was detected in: The pol/S region (63 bp amplicon) for six patients, the pol/S region (217 bp amplicon) for three patients, the DR2/X region for eight patients, the DR1/X region for nine patients, and the core region for eight patients.

We and other have shown that the targeted DNA sequence is more readily detectable by PCR when the background DNA in the reaction is reduced [17, 22–26]. Since most of the detectable HBV DNA in urine is less than 1 kb, it was of interest to determine if HBV DNA could be detected in the urine of patients with low serum viral loads, or below the limit of detection in total urine DNA. Hence, total urine DNA from the remaining 47 patients was fractionated to obtain LMW DNA fraction and subjected to the four short (< 100 bp) HBV DNA assays. From this, an additional nine urine samples were found to contain HBV DNA specific for the DR2/X region and six samples specific for the DR1/X assay (Fig. 2).

### Association between urine HBV DNA and clinicopathological variables

HBV DNA was detected in the serum from 59 of the 60 CHB patients. Sixteen of 25 urine samples with high viral load (> 10^5^ IU/mL) and 11 of 34 urine samples with low viral load (< 10^5^ IU/mL) contained detectable HBV DNA in urine. By Fisher’s exact test, HBV DNA in urine is significantly associated with high serum viral load (*P* = 0.0197). Fifty-eight of the 60 patients in this study were HBsAg positive and 17 of 60 were HBeAg positive. Twelve of 27 (44.44%) patients with detectable HBV DNA in urine were HBeAg positive as compared to only 5 of 33 (15.15%) patients with no detectable HBV DNA in urine that were HBeAg positive, suggesting a significant association between active viral replication and HBV DNA in urine by Fisher’s exact test (*P* = 0.0203). Twelve of 17 (70.58%) of HBeAg positive patients had detectable HBV DNA in urine, whereas only 15 of 43 (34.88%) HBeAg negative patients had detectable HBV DNA in urine. There was no correlation between urinary HBV DNA and age, gender, or AST and ALT levels by Kruskal-Wallis test (*P* > 0.05). While one patient with full length HBV DNA had hematuria and albuminuria, not every patient with compromised kidney barrier/function had detectable HBV DNA in their urine. Statistical analysis was performed using a Mann Whitney U test to assess the association between the number of HBV DNA fragments detected (5 regions of the HBV genome) and liver pathology (51 chronic hepatitis B, 7 cirrhosis). The 2 samples from HCC patients were not included in this analysis. We found no statistically significant liver disease association (*p* = 0.170) with the number of HBV DNA fragments detected.

## Discussion

This study investigated the integrity of HBV DNA in the urine of 60 CHB patients, (including 11 of 60 patients with serum viral loads in a range of 10^7^–10^8^ IU/mL), by employing 6 different PCR assays that target unique regions of the viral genome. Only one patient (ID #1), with an indication of compromised renal barrier (and blood in the urine), was thought likely to contain full-length HBV DNA in the urine. In the remaining patients with detectable HBV DNA in the urine, the DNA was found to be fragmented. We thus conclude that urine from CHB patients with healthy kidney function should not contain full-length HBV DNA, and therefore should not be infectious. To our knowledge this is the first study to investigate the integrity of HBV DNA in a comprehensive manner by examining multiple locations of the HBV genome.

While there are other methods available for detection of HBV virus (such as southern blot or protein analysis), we chose a PCR approach because it is the most sensitive method available. Since we were unable to detect full-length HBV DNA by PCR, less sensitive methods are unlikely to detect intact, infectious viral particles. Although HBV susceptible cell lines (such as HepG2-NTCP cells) are available for direct infectivity testing with cell culture derived virus, they require high multiplicity of infection (MOI > 100) [27, 28]. It has been reported that serum-derived virus is difficult to infect NTCP cells for unknown reasons. For urine samples that are negative for full-length HBV DNA by PCR (with a limit of detection of 20 copies per mL of urine), sufficient levels of virus are simply not available to accurately test cell-culture infectivity. Thus, the infectivity assay was not performed in this study.

Viruria (virus in urine) has only been suggested in viral infections of kidney cells, such as cytomegaloviruses [29], coxsackie viruses [30], adenoviruses [31] etc [32]. Hence, it is not surprising that the full-length HBV virus was is not detected in the urine of the CHB patients, even when serum viral loads reach 10^8^ IU/mL, unless there is blood contamination or compromised kidney function. Interestingly, case ID’s #2 and #5 showed impaired renal structure with high serum viral load but no sign of blood contamination, which seems to suggest that blood contamination might be the source of full-length HBV DNA detectable in the urine of Pt. ID #1. Urine from Pt. ID #32 (with a serum viral load of 1.7 × 10^4^ IU/mL) and Pt. ID #58 (with a serum viral load of 20 IU/mL) were also contaminated with blood, but no HBV DNA was detected in urine suggesting that high serum viral load might be necessary to have full-length HBV DNA detected above the limit of detection in urine.

According to Knutsson et al. [11], HBV DNA was detected (by PCR) in serum from 46 of 56 CHB patients (82%) and in urine from 28 of 56 patients (50%). Most HBeAg (+) patients had HBV DNA detectable in urine (91%). Robert et al [14] detected HBV DNA from urine in 32% (47/147) of patients with CHB and in 60% (39/65) with HBeAg positive status. In addition to PCR detection, molecular hybridization methods have been used to detect HBV DNA from urine in about 55% of chronic HBV cases that are positive for HBeAg [12]. In the present study, we found that 45% (27/59) of CHB patients with detectable HBV in serum contained detectable HBV DNA in urine. Similar to previous studies [11, 12, 14], 70.58% (12/17) of HBeAg positive patients had detectable HBV DNA in urine, suggesting that active viral replication is associated with HBV DNA in urine.

Interestingly, the HBV DR2 (*n* = 17) and DR1 (*n* = 15) regions were more frequently detected (of the HBV qPCR assays) as compared to the pol/S (*n* = 6) and core region (*n* = 8) of the HBV genome. One would expect a similar frequency of detection regardless the region of the genome if fragmentation of the DNA were random. Our data suggest that the fragmentation of the genome or preservation of the fragmented HBV DNA could be different from region to region. Our previous studies have suggested the LMW DNA in urine is mostly derived from circulation and from cells undergoing cell death. Most of the HBV DNA detected in urine is also detected in the LMW DNA fraction, which suggests the HBV DNA detected in urine is predominantly cell-free DNA likely derived from apoptotic, infected hepatocytes.

## Conclusions

In conclusion, this study of HBV DNA integrity in urine suggests that urine is likely not infectious, except in cases of compromised renal structure or blood contamination. Although an infectivity study was not conducted here, the absence of detectable, full-length HBV genomic DNA in urine suggests the risk of HBV infection from urine is very unlikely. This is of particular importance for infection control guidelines to prevent HBV transmission.

## Additional file

Additional file 1: Urine DNA analysis by qPCR assays targeting multiple locations in the HBV genome. Raw data for Fig. 2. (XLSX 18 kb)

## Abbreviations

ALT: alanine transaminase
AST: aspartate transaminase
CHB: Chronic HBV infection
CRP: C-reactive protein
HBeAg: Hepatitis B envelope antigen
HBsAg: Hepatitis B surface antigen
HBV: Hepatitis B virus
HMW: HIgh molecular weight
LMW: Low molecular weight
qPCR: Quantitative PCR
